# Bedtime Routine Characteristics and Activities in Families with Young Children in the North of England

**DOI:** 10.3390/ijerph18178983

**Published:** 2021-08-26

**Authors:** George Kitsaras, Michaela Goodwin, Julia Allan, Michael P. Kelly, Iain A. Pretty

**Affiliations:** 1 Dental Health Unit, Division of Dentistry, The University of Manchester, Manchester M15 6SE, UK; michaela.goodwin@manchester.ac.uk (M.G.); iain.a.pretty@manchester.ac.uk (I.A.P.); 2 Dental Health Unit, Williams House, Manchester Science Park, Manchester M15 6SE, UK; 3 Institute of Applied Health Sciences, University of Aberdeen, Aberdeen AB24 3FX, UK; j.allan@abdn.ac.uk; 4 Department of Public Health and Primary Care, University of Cambridge, Cambridge CB1 8RN, UK; mk744@medschl.cam.ac.uk

**Keywords:** child, family, health, parenting, wellbeing

## Abstract

Bedtime routines have been shown to have significant associations with health, wellbeing and development outcomes for children and parents. Despite the importance of bedtime routines, most research has been carried out in the United States, with little information on bedtime routine characteristics and activities for families in other countries such as the United Kingdom and England in particular. Additionally, little is known about the possible effects of weekends vs. weekdays on the quality of bedtime routines. Finally, traditional, retrospective approaches have been most used in capturing data on bedtime routines, limiting our understanding of a dynamic and complex behaviour. The aim of this study was to explore bedtime routine characteristics and activities in families in the North of England with a real-time, dynamic data collection approach and to examine possible effects of weekend nights on the quality of bedtime routines. In total, 185 parents with children ages 3 to 7 years old provided data around their bedtime routine activities using an automated text-survey assessment over a 7-night period. Information on socio-economic and demographic characteristics were also gathered during recruitment. A small majority of parents managed to achieve all crucial elements of an optimal bedtime routine every night, with 53% reporting brushing their children’s teeth every night, 25% reading to their children every night and 30% consistently putting their children to bed at the same time each night. Results showed significant differences between weekend (especially Saturday) and weekday routines (*F*(1, 100) = 97.584, *p* < 0.001), with an additional effect for parental employment (*F*(1, 175) = 7.151, *p* < 0.05). Results highlight variability in bedtime routine activities and characteristics between families. Many families undertook, in a consistent manner, activities that are closely aligned with good practices and recommendations on what constitutes an optimal bedtime routine, while others struggled. Routines remained relatively stable during weekdays but showed signs of change over the weekend. Additional studies on mechanisms and elements affecting the formation, development and maintenance of bedtime routines are needed alongside studies on supporting and assisting families to achieve optimal routines.

## 1. Background

Past research, starting from Bronfenbrenner’s ecological model, and more recent studies, including research by Fiese [1], have all highlighted the importance of repetitive, predictable proximal processes for children’s development. Activities that occur at the same time each night, following a similar, familiar pattern, actively promote a safe and reassuring environment for children. Predictability and safety are vital components for family routines and increase compliance with a given task. Bedtime routines can be described as a recurrent, dynamic and common set of activities that take place roughly the hour before children go to bed [1]. Previous studies on bedtime routines have shown the importance of bedtime routines in terms of quality of sleep [2,3], dental health [4,5], school performance [6] and school readiness [7,8], psycho-social and emotional development [9], overall family functioning and parental stress and confidence [1,8]. Moreover, intervention studies have shown that it is possible to intervene and alter these routines with subsequent benefits for children and parents alike [10].

Bedtime routine activities can encompass a diverse range of interactive, non-interactive and hygiene activities [10]. These activities include: dietary habits such as bottle feeding/drinks, snacks before bed; bath and other hygiene, including oral hygiene, behaviours; proactive, prosocial and positive parent–child interactions, such as being rocked, singing songs, listening to music, massage and playing; reading or sharing books and storytelling before bed, and finally, use of electronic devices including watching TV, playing or interacting with a gaming console, tablet, mobile phone or computer before bed [10]. These activities can be further categorised into adaptive (i.e., hygiene behaviours, reading books) and maladaptive (i.e., snacks/drinks, watching TV/using electronic devices) [11]. These activities around bedtime routines are not consistent throughout childhood, with necessary adaptations as children grow older, for example, rocking will eventually stop as the child moves from infancy to toddlerhood and beyond, while other activities, such as watching TV/using electronic devices, may become more prevalent as a child ages [10].

An optimal bedtime routine for young children, ages 2–8 years old, should: (a) be consistent throughout the week following the recommended sleep hours for each age group, (b) include tooth brushing and other positive, beneficial hygiene behaviours such as a bath before bed, but not necessarily every night, (c) avoid drinks (such as bottle feeding) and snacks before bed, excluding water and unflavoured milk that can actually form part of the routine itself, (d) minimise the use of electronic devices, including television, around and during bedtime, and finally, (e) include book reading and book sharing activities including storytelling before bed [2,4,8,10,12]. Despite differences in developmental processes, expert agreement has showcased the great overlap in bedtime routine characteristics for children between ages of 2 and 8 resulting in important opportunities for exploring these age groups together [11].

Despite growing evidence for the importance of bedtime routines, there is a limited understanding of the characteristics and prevalence of bedtime routines across different countries and across populations of varying socio-economic and demographic composition [10]. In their systematic review, Mindell and Williamson [10] highlighted the issue of limited studies around bedtime routine characteristics. From available research, it is known that most families have some sort of routine around bedtime, but little is known about what takes place during that routine [10]. Research in the United States has shown differences in the implementation and characteristics of bedtime routines between families of different ethnic and socioeconomic backgrounds [12]. However, similar studies are lacking on a global scale, including in the United Kingdom (UK), and in particular, England [13]. The same review identified the additional need for longitudinal studies that examine bedtime routines in more detail rather than through the utilisation of one-off retrospective measures. Most studies within bedtime routines deployed retrospective, paper-based questionnaires to capture data. Retrospective data collection can increase the likelihood of recall bias, while paper-based approaches allow very little room for dynamic data capturing, especially for a dynamic and complex issue such as bedtime routines. Finally, a lack of studies examining the possibility of a weekend effect on the quality of bedtime routines further limits our understanding on how to best support families to achieve optimal routines. With potentially important differences in the quality of routines throughout the week, exploring these possible changes is important.

### Aims

Overall, the aim of this study is to examine bedtime routine characteristics and activities in families with young children in the North of England in order to offer some preliminary yet vital information of what unfolds during bedtime. Additionally, this study aims to utilise a real-time, dynamic approach for data collection to potentially overcome limitations found in retrospective, paper-based data collection approaches. The study aims to explore possible differences in the quality of bedtime routines between weekdays and weekends, and finally, examine the role of demographic and socio-economic characteristics on the quality of bedtime routines.

## 2. Methods

A cross-sectional study exploring bedtime routines characteristics in families with young children using a real-time, dynamic data collection approach was completed between February and July 2018. The study in its entirety, including consent forms and all study materials, was approved by the Health Research Authority (Integrated Research Application System (IRAS) ID: 238552).

### 2.1. Sample and Recruitment

In total, 200 parents were recruited with children between the ages of 3 to 7 years of age. There was an overall consent rate of 65%, with 308 people approached in total. Inclusion criteria included: (a) ability to speak and comprehend English, (b) owning a working mobile phone, not necessarily a smartphone, and (c) having children between the ages of 3 and 7 years old. Eligible participants were firstly identified by relevant staff at health and dental centres in the North of England, and initial contact was then made by a researcher who provided information about the study. Compensation (£10 shopping vouchers) was provided at the end of the study in the form of online shopping vouchers.

### 2.2. Data Collection

Data collection was completed by one parent, with demographic information reflecting data related to the parent who completed the questionnaire. Data collection took place over a 7-night period in order to capture a wider range of data points per participant, including fluctuations in bedtime routines occurring between weekdays and weekends. In line with a previous study on bedtime routines [8], an automated text-survey was utilised to capture data on bedtime routines. In a previous study [8], the automated text-survey was successfully deployed to capture data on bedtime routines in families with young children, with parents providing positive feedback on the use of the survey. The automated text-survey, identical to the one used in a previous study [8], included both open- and close-ended questions (a breakdown of questions asked on a nightly basis is presented in Appendix A Table A1). Questions covered all areas associated with an optimal bedtime routine, such as: time consistency (what time child went to bed), dietary habits before bed (including drinks and snacks before bed), oral hygiene behaviours (i.e., toothbrushing), use of electronic devices (including watching TV) before bed and book reading/sharing (or storytelling) before bed. During recruitment, parents agreed on a predetermined time for receiving the survey, aiming for data collection just after their routine was completed. Each night, parents could delay, defer or decide not to complete the survey. The survey was delivered to their mobile phone. Secure servers and secure online platforms were used to develop and deliver the survey to parents’ phones. All information sent back by parents was anonymised and securely kept, minimising risk to their personal information.

Information on socioeconomic and demographic characteristics were collected during recruitment through the completion of a brief demographics form. Index of Multiple Deprivation (IMD) scores, a commonly used metric of deprivation in England, were calculated based on the information provided by participants. Higher scores reflect higher levels of deprivation.

### 2.3. Data Analysis

Each night’s routine was assessed based on available guidelines on what constitutes an optimal bedtime routine. As with a previous study [8], a score of 1 was assigned to each of the 5 selected activities (1 point for undertaking the activity and 0 points for not undertaking the activity, with reverse scoring for dietary habits before bed and use of electronic devices). Average scores for the 7 nights were calculated and used for further analyses. High scores indicate better bedtime routines. Data were descriptively analysed in order to examine frequency and prevalence of bedtime routines and activities. Since not all families responded to every night of the assessment, percentage scores were used instead of raw scores to reflect frequencies. All data were analysed using SPSS v.25 (IBM Corp. 2017, Armonk, NY, USA). Multiple comparisons were used to examine within- and between-subject variance regarding a participant’s bedtime routine scores over the study period and the possibility of a ‘weekend effect’ impacting the quality of bedtime routines. Demographic and socioeconomic information were used to examine possible effects on overall quality of bedtime routines using one-way analysis of variance (ANOVA). Finally, in terms of missing data, two analyses were calculated: one by excluding missing data and another one with median imputation replacing missing data. No significant differences were found, and therefore, it was determined that missing data did not significantly affect the results. All data presented are based on the original dataset, with missing data excluded from analyses.

## 3. Results

### 3.1. Sample Characteristics

Out of 200 parents, 185 parents completed data collection (92% retention rate). Parents had a mean age of 34.6 (SD = 5.01), with the youngest participant being 25 years of age and the oldest 46 years of age. Most participants were female, with only 13% (*n* = 24) being male. Table 1 summarises key demographic information. Due to the areas where recruitment was undertaken, there was a significant proportion of Asian/British-Asian participants, reflecting the ethnic composition of recruitment sites.

### 3.2. Response Rates

All but one participant replied to at least 3 out of 7 nights. The participant who replied for only 2 nights was removed from subsequent data analyses due to a low number of replies. From the remaining 184 participants, most of them (*n* = 100) replied to every night of the assessment while 56 replied to 6/7 nights, 16 replied to 5/7 nights, 9 replied to 4/7 nights and finally, only 3 participants replied to 3/7 nights.

### 3.3. Overview of Bedtime Routine Characteristics and Activities

In total, five different bedtime routine activities were measured as part of this study. These are presented as percentage of participants who completed the activity based on their replies on a nightly basis. Table 2 summarises the overview of bedtime routine characteristics and activities. In general, a small majority of participants reported brushing their children’s teeth every night (53%). Only 4.4% of participants reported completely avoiding snacks or drinks the hour before bed, while 36 (19.7%) reported having snacks or drinks every single night before bed. From those allowing food and/or drinks the hour before bed, 44.3% gave their children water or unflavoured milk, 16.9% allowed consumption of fruit or vegetables, while 14.3% allowed sugary or savoury snacks, including chocolate, crisps or soft drinks. Finally, a total of 25.1% of participants read to their children every night of the week, 9.3% never read or shared a book with their children during the course of the study, only 8.2% (16 out of 184) did not allow use of electronic devices at all the hour before bed, while around 30% of parents achieved time consistency (i.e., within the hour of the time that the child went to bed the night before) in getting their children to bed every night.

A repeated measurements ANOVA was completed to examine if there was an exposure effect based on how many days a participant completed the text-survey. This looked to examine any differences between the first night of the assessment vs. the remaining nights to determine changes in bedtime routine scores overtime. Due to the nature of recruiting participants, there were differences regarding the start of data collection, for example, some participants might have started their data collection on a Monday (night 1), while someone else might have started on a Friday (night 1). A significant difference was observed for night 1 vs. night 2 (*F*(1, 92) = 5.194, *p* < 0.001), night 1 vs. night 4 (*F*(1, 92) = 6.186, *p* < 0.001) and night 1 vs. night 5 (*F*(1, 92) = 7.886, *p* < 0.001). On the contrary, there was no significant change between night 1 vs. night 3, night 1 vs. night 6 and night 1 vs. night 7. The results of the analysis showed no clear exposure effect, even though there were significant differences between the first night of completing the survey and other nights later in the week. Effects of demographics and socioeconomic characteristics (education level, IMD score, ethnicity, employment status and household type) were included in this analysis, with no significant modifications regarding observed differences.

### 3.4. Examining Weekend Effects on Quality of Bedtime Routines

Examining possible effects of weekend nights compared to weekday nights was conducted using repeated measures ANOVAs. Possible changes in overall bedtime routines’ quality were examined by comparing Saturday and Sunday scores with scores on different nights of the weeks. The results of this analysis showed that across all other nights (Monday, Tuesday, Wednesday, Thursday, Friday), there were significant differences in the overall bedtime routine scores when compared to Saturday and Sunday scores. Table 3 presents the results of this analysis comparing Saturday and Sunday results to the rest of the week. Effects of demographics and socioeconomic characteristics were included in this analysis, with no significant modifications regarding observed differences.

### 3.5. Examining Effect of Demographic and Economic Characteristics on Quality of Bedtime Routines

A series of one-way ANOVAs was calculated to examine possible effects of demographic and socioeconomic characteristics affecting the overall quality of bedtime routines. Employment status, education, age of parent, age of children, number of children, ethnicity, household type and IMD scores were included in this analysis. The results of the analysis showed that only employment had a significant effect on overall quality of bedtime routines (*F*(1, 175) = 7.151, *p* < 0.05). Post-hoc comparisons between the different types of employment showed that there were significant differences in mean bedtime routine scores between full-time vs. part-time employed parents and full-time vs. stay at home parents. Parents employed full-time were shown to have, on average, a lower (and therefore less optimal) bedtime routine score (mean = 2.7, SD = 0.64) compared to parents working part-time (mean = 3.23, SD = 0.89) and stay at home parents (mean = 3.23, SD = 0.74). Based on these results, it appears that both stay at home parents and parents working part-time achieved better overall bedtime routine scores when compared to those parents who work full-time.

## 4. Discussion

So far, little is known about bedtime routines in families with young children living in the North of England, the possibility of a weekend effect on the quality of bedtime routines and the impact of socioeconomic and demographic factors that can affect bedtime routines. Similar to other studies [10], key results from this study show that all families implement one form of routine around bedtime each night, and while some families manage to achieve optimal routines, other struggle. Additionally, this study offered some preliminary evidence of a difference in the quality of bedtime routines between weekdays and the weekend, with routines being significantly worse in the latter period. Finally, employment status appears to affect the quality of bedtime routines.

### 4.1. Importance of Bedtime Routine Activities in Overall Child Wellbeing and Development

The present study simply examined prevalence, frequency and characteristics of bedtime routines in families with young children. No measurements on the impact of those routines on areas such as sleep, parental mood and family functioning were included. Therefore, there is no basis for causal or associative connections between the results of this study and the impact of optimal vs. suboptimal bedtime routines. However, and based on available data regarding bedtime routines, successful and consistent implementation of an optimal bedtime routine can have important, long-term benefits for children and their families alike. Good oral hygiene behaviours, including frequent tooth brushing before bed as well as avoidance of food and/or drinks (excluding water and unflavoured milk) before bed, can lead to overall better dental health [12,14]. Lack of dental problems early in life can then have positive implications for a child’s overall development, leading to avoidance of dental extractions, dental pain, loss of sleep due to dental pain and missing days in school [15,16,17]. Apart from dental health, good dietary habits the hour before bed have shown important associations with obesity rates [18,19]. In this study, it was found that just over half (53%) of parents managed to consistently brush their children’s teeth every night, with around 1.1% never reporting brushing their children’s teeth over the course of the data collection process. Moreover, most parents (175 out of 184) allowed either snacks or drinks other than water or unflavoured milk the hour before bed, resulting in problematic dietary habits for their young children.

Book reading, sharing a book with children as part of the bedtime routine or simply storytelling can promote child literacy, improve school performance and enhance school readiness in young children, with subsequent possible implications in later achievement and attainment [20,21]. Based on the results of this study, around 10% of parents never read to their children before bed, while another 36.1% read or shared a book with their children for less than half of the nights observed. Book reading or sharing a book with a young child as part of his/her bedtime routine is a crucial element for successful later development, and therefore it is a behaviour that needs proactive and consistent participation from parents.

Having a consistent, appropriate time that children go to bed could aid in achieving adequate hours of sleep. In addition, maintaining the routine around a stable and protected time each night can further reinforce the formation of habits and rituals for the family and the child [22]. Finally, optimal routines can lead to better family functioning with less behavioural issues (i.e., tantrums, bedtime resistance), while enhancing parent–child relationships and interactions and improving parental socio-emotional wellbeing [23,24,25]. In terms of time consistency, this is the only area where a larger majority of parents either achieved it every night or tried and achieved it for at least half of the nights they replied to the text-surveys. Overall, 67.2% of parents managed to achieve consistency in getting children to bed, with only 5.5% or 10 out of 184 people failing to do so every single night of the data collection period.

The only activity where results presented a more mixed picture between those with overall optimal and those with overall sub-optimal bedtime routines was use of electronic devices before bed. Use of electronic devices at some point during the week was reported from 92% of the sample. Based on available data collected from the automated text-survey, it is not possible to distinguish what type of engagement with electronic devices those 92% had. Use of electronic devices is a broad subject, ranging from watching TV to reading an e-book on a portable reader. The recent rise in access rates to electronic devices from a younger age and time spent in front of a screen has prompted a more robust look at possible effects and implications. Recent studies have shown that prolonged exposure to electronic devices can lead to overall issues around development, with particularly important implications around sleep as well as cognitive, educational and behavioural development [26]. With a possible important link between the use of electronic devices and overall child wellbeing and development, it is important to further explore the types of interactions and uses of those devices in the context of bedtime routines.

Finally, results showed evidence of a “weekend” effect, where the quality of bedtime routines deteriorates closer to and around the weekend when compared to weekdays or school-nights. This is an interesting and previously non-reported finding. Anecdotal evidence could suggest that parents and families at large might alter their behaviours between weekday and weekend nights, with Friday and Saturday nights commonly referred to as “weekend” nights. The mere labelling of some nights as school-nights and other nights as weekend nights can provide sufficient justification for the creation of cognitive, at first, and later, practical and behavioural modifications, and altered expectations on what children are supposed to do and not do. There is no specific evidence base for this hypothesis, but the conscious and sub-conscious power that simple labels might have on people’s behaviours and expectations has been previously observed [27]. It is also possible that Saturday (and the weekend at large) activities are distinct, with each family engaging in specific activities with a specific meaning for them, but this needs to be further explored in subsequent studies.

### 4.2. Demographic, Socioeconomic Factors and Implications for Bedtime Routines

Previous studies have reported differences in prevalence and frequency of bedtime routines between white and non-white samples, as well as samples from predominately Asian countries and regions [28,29]. Despite prior evidence of significant differences in the quality and frequency of bedtime routines amongst different ethnic groups [10], this study failed to replicate this difference. Additionally, with a long-established misrepresentation of minority ethnic groups in health research [30], it is crucial for studies to be able to include and report on communities that are routinely overlooked. Examination of diverse populations can allow for better and more inclusive findings for a recurrent family activity that transcends sociodemographic and ethnic boundaries. This study utilised a diverse sample, reflecting the composition of local communities where recruitment took place.

### 4.3. Limitations

Risk of bias, especially desirability bias, is probably the most important possible limitation of this study. As with every self-reported measure, it is difficult to control for the effects of this type of bias since it is the responsibility of the participant to reply with accuracy and honesty, with no means of guaranteeing the accuracy of their responses. The development and deployment of the text-survey assessment of bedtime routines aimed at reducing effects of desirability bias by introducing a more intrinsic approach that utilised a rapid and more automated response pattern from participants. Additionally, missing data is another limitation, despite attempts to manage the implication of missing data on subsequent analyses. Lack of complementary measurements, especially around children’s sleep, further limits our overall understanding on the quality of bedtimes and how they can affect an important area of child wellbeing and development. The bedtime routines survey used in this study was also not validated, despite being used successfully in previously published research with a similar population. Finally, the lack of information on both parents and the focus on one parent specifically hinders our analyses, since there is no clear, holistic picture of family life and family dynamics. More detailed and in-depth demographic questions would have allowed for a better exploration of family structures and how they might have affected bedtime routines.

## 5. Conclusions

This study examined bedtime routines in families with young children. Some families appear to follow what is considered an optimal bedtime routine for most of the week, with a slight, previously unreported, change in routines over the weekend. With bedtime routines occupying a central role in family functioning, it is important to quantify how routines manifest themselves on a day-to-day basis in order to progress our understanding of this crucial family behaviour. Despite some shortcomings, this work has the potential to inform future directions of research in this area while also unearthing important findings on routines across the socioeconomic, ethnic and demographic divide.

## Figures and Tables

**Table 1:** Demographic characteristics of the sample.

Item	Result
Children per family	
- 1 child	65% (*n* = 120)
- 2 children	30% (*n* = 55)
- 3 children	5% (*n* = 10)
Child gender	
- Female	43.2% (*n* = 80)
- Male	56.8% (*n* = 105)
Ethnicity	
- White	48.1% (*n* = 89)
- Black/British-Black/Caribbean	12.4% (*n* = 23)
- Asian/British-Asian	39.5% (*n* = 73)
Index of Multiple Deprivation (IMD) score	
- Mean (SD)	41.83 (SD = 16.43)
Education (parents)	
- High school graduates	63.8% (*n* = 118)
- Post-high school graduates (i.e., vocational training, colleges, non-University degrees)	29.2% (*n* = 54)
- University graduates	7% (*n* = 13)
Employment (parents)	
- Stay at home parents	40.5% (*n* = 75)
- Part-time work	29.7% (*n* = 55)
- Full-time work	19.5% (*n* = 36)
- Students	7% (*n* = 13)
- Unemployed	3.2% (*n* = 6)
Household type	
- Two-parent household	85% (*n* = 157)
- One-parent household	15% (*n* = 28)
- Multigenerational households (out of 185)	10% (*n* = 18)

IMD average scores calculated based on the Index of Multiple Deprivation used in England to assign households a deprivation score based on their address. The higher the score, the more deprived an area/household.

**Table 2:** Overview of each targeted activity as part of the bedtime routine.

Activity	Achieved Every Night		Never Performed		Achieved at Least 50% of Nights Replied		Achieved Less than 50% of Nights Replied		*n* Total % Total	
	N	%	N	%	N	%	N	%		
Tooth brushing before bed	98	53.2	2	1.1	61	33.1	23	12.5	184	100
Diet before bed (allowing snacks and/or drinks)	36	19.5	9	4.9	63	34.2	76	41.3	184	100
Book reading before bed	47	25.5	17	9.2	54	29.3	66	35.8	184	100
Using electronic devices before bed (allowing use)	27	14.7	16	8.7	80	43.5	61	33.1	184	100
Time consistency in getting child to bed	57	31.0	10	5.4	67	36.4	50	27.1	184	100

**Table 3:** Examining weekend effects on quality of bedtime routines.

Weekend/Weekdays		M (SD) Night 1	M (SD) *	F	Sig.
Saturday	Sunday	2.27 (1.12)	2.74 (0.89)	78.155	0.132
	Monday		3.37 (1.00)	97.584	<0.001
	Tuesday		3.64 (0.94)	106.452	<0.001
	Wednesday		3.47 (1.39)	103.318	<0.001
	Thursday		3.19 (1.22	73.818	<0.001
	Friday		3.27 (0.94)	84.167	<0.001
**Weekend/Weekdays**		**M (SD) Night 1**	M (SD) *	**F**	**Sig.**
Sunday	Monday	2.74 (0.89)	3.37 (1.00)	32.314	<0.001
	Tuesday		3.64 (0.94)	0.789	<0.001
	Wednesday		3.47 (1.39)	103.318	<0.001
	Thursday		3.19 (1.22	1.474	<0.001
	Friday		3.27 (0.94)	6.678	<0.001
	Saturday		2.27 (1.12)	2.752	0.132

## Data Availability

The datasets used and/or analysed during the current study are available from the corresponding author upon reasonable request.

## Appendix A

**Table A1:** Open and closed-ended questions sent to participants each night using the automated text survey system. Details: A list of questions parents received each night as part of the study. The questions were sent to parents’ mobile phones each night for the duration of the study.

Questions Asked Each Night during the Study	
Q1	HELLO! This is the research team! We are just going to ask you some questions about tonight’s bedtime. Is this a good time? Reply “Yes” or “No”
Q2	How would you rate tonight’s bed time routine from 0 (many problems, worst routine for a while) to 5 (best routine ever!). Please rate from 0 to 5.
Q3	What time did your child go to bed? If he/she is still awake, please reply “Awake” or “Not yet”.
Q4	Who was involved in tonight’s routine? Mum, Dad, Both parents or someone else? Please reply “Mum”, “Dad”, “Both” or “Other”.
Q5	Did your child eat or drink anything the hour (i.e., not his/her dinner) before bed? Please reply “Yes” or “No”.
Q5.a.	What was it? Please briefly describe what your child ate and/or drank the hour (i.e., not dinner) before bed (i.e., glass of milk, chocolate, fruit etc.)
Q6	Were your child’s teeth brushed tonight? Reply “Yes” or “No”
Q6.a.	Who brushed them? Please reply “Child” or “Parent”, or if you helped your child brush their teeth then reply “Together”
Q7	Did your child play video games, watch TV or use any electronic devices (incl. mobile phones, tablets etc.) the hour before bed? Please reply “Yes” or “No”
Q8	Did you read a story to your child before bed? Please reply “Yes” or “No”
Q9	Thank you! We will be in contact soon. Remember if you want to leave the study, and receive no more text-messages please text “Leave”, otherwise we are all done.
